# Retraction Note: Inhibition of airway inflammation in a cockroach allergen model of asthma by agonists of miRNA-33b

**DOI:** 10.1038/s41598-019-53536-0

**Published:** 2019-11-12

**Authors:** Ruichao Niu, Xuping Xiao, Bin Liu, Yunqiu Li, Yu zhong, Lijuan Ma

**Affiliations:** 1 Department of Respiratory Medicine, Xiangya Hospital, Central South University, Changsha, 410008 P.R. China; 2 Department of Otolaryngology Head and Neck Surgery, Hunan Provincial People’s Hospital, The First Affiliated Hospital of Hunan Normal University, Changsha, 410008 P.R. China

Retraction of: *Scientific Reports* 10.1038/s41598-017-07882-6, published online 07 August 2017

The authors retract this article.

The authors discovered that an anti-tryptase antibody used in this study was out of date at the time of original experiments. They repeated the experiments depicted in Figures 3A, 3C, and 7C with a fresh antibody, but were not able to replicate the results. The authors did repeat Western blot experiments shown in Figure 6C, as well as immunofluorescence experiments shown in Figure 1D, but were also unable to replicate these results, and the original data for these figures could not be located. Since the affected figures report some of the main results of the article, the authors are unable to guarantee its conclusions and decided to retract the paper.

All authors agree with a retraction.

